# Surgical management of ulnar styloid fractures: comparison of fixation with anchor suture and tension band wire

**DOI:** 10.1186/s13018-020-01795-3

**Published:** 2020-07-21

**Authors:** Alvin Chao-Yu Chen, Yi-Hsuan Lin, Chun-Jui Weng, Chun-Ying Cheng

**Affiliations:** Bone and Joint Research Center, Department of Orthopaedic Surgery, Chang Gung Memorial Hospital–Linkou and Chang Gung University College of Medicine, 5th, Fu-Shin Street, Kweishan District, Taoyuan, 333 Taiwan, Republic of China

**Keywords:** Ulnar styloid; Distal radioulnar joint (DRUJ); Distal radius fracture (DRF), Suture anchor, Ultra-braid suture, Tension band wire

## Abstract

**Background:**

Limited reference is available regarding surgical management in symptomatic ulnar styloid fractures with small bony avulsion. The study goal is to report the surgical outcomes using anchor suture fixation with comparison to traditional tension band wire fixation.

**Methods:**

We retrospectively reviewed the medical records in patients who underwent surgical repair for unilateral ulnar styloid fractures with distal radioulnar instability between 2004 and 2017. A total of 31 patients were enrolled including two kinds of fixation methods. Anchor suture fixation plus distal radioulnar joint pinning was performed in ten patients with tiny avulsion bony fragments (group A); tension band wire fixation was performed in 21 patients with big styloid fracture fragments (group B). Patient characteristics and 2-year treatment outcomes were compared between two groups based on Mayo Modified Wrist Score (MMWS); Quick Disabilities of the Arm, Shoulder, and Hand (QuickDASH); visual analog scale (VAS), and surgical complication. Descriptive statistics were used for calculation of key variables; a *p* value of < 0.05 was considered statistically significant.

**Results:**

Based on Gaulke classification, there were five subtypes in group A and three subtypes in group B. Incidence of concomitant distal radius fractures was significantly higher in group B; other patient characteristics including age, sex, injury side, and time to surgery showed no significant difference. Outcome assessment regarding MMWS, QuickDASH, and VAS was comparable between two groups. Bone-related complications including nonunion, DRUJ subluxation, and styloid resorption were analyzed; the difference was not significant. Incidence of implant-related complications including migration and secondary removal surgery was significantly higher in group B (*p* = 0.021).

**Conclusion:**

Surgical fixation in symptomatic ulnar styloid fractures yields comparable treatment outcomes in both fracture patterns. Implant-related complication with secondary removal surgery is more common in tension band wire group. Anchor suture fixation is a feasible option for tiny styloid avulsion fragments with limited surgical complication.

## Background

Being a pivotal point for rotation of forearm and load transmission of wrist, traumatic insults on the distal ulna usually affect both distal radioulnar joint (DRUJ) and ulnar-carpal joint [1]. Treatment modalities are aimed to restore anatomic structure to facilitate functional recovery. Ulnar styloid process is an elongated part distal to ulnar head and responsible for insertion of triangular fibrocartilage complex (TFCC) at the base [2]. Fractures of the ulnar styloid are commonly mentioned with the distal radius fracture (DRF) [3]; treatment options and prognosis are generally correlated with the presence of DRUJ instability [4, 5]. For styloid base fracture with big avulsion fragment, tension band wiring or plate fixation is commonly adopted [6, 7]. However, there was limited reference regarding surgical management for small bony avulsion except for simple excision of the styloid fragment in cases with symptomatic nonunion [8]. Currently, technical refinement and implant evolution allow fixation of tiny avulsion fragments instead of excision to obtain bony healing and joint stability [9–11]. The purpose of this study is to present a novel surgical technique using suture anchors to fix symptomatic ulnar styloid fractures with small bony fragments and to report treatment outcomes.

## Methods

### Patient demographics

Ethics committee approval from our hospital was obtained (IRB 201800834B0) to review the medical records in patients receiving surgical fixation for unilateral ulnar styloid fractures between 2004 and 2017. A total of 31 patients were enrolled including 20 male and 11 female patients. All were displaced ulnar styloid fractures with distal radioulnar instability and were divided into two groups based on fixation methods. Group A consisted of patients with small bony avulsion (six patients), comminuted fractures (two patients), and revision cases (two patients) that underwent anchor suture fixation for the styloid fragment followed by percutaneous pinning of the distal radius and ulna with sparing the DRUJ. The two revision cases had failed to previous surgery with screw fixation. Group B comprised of patients of ulnar styloid base fractures with big bony fragments, which was fixed with tension band wire. There were 10 patients in group A and 21 patients, group B. Patients characteristics were compared in Table 1. Concomitant DRFs were noted in 9 patients (1 in group A and 8 in group B) and underwent open reduction and plate fixation. Radiographic classification based on Gaulke classification [12] was shown in Table 2.

**Table 1:** Demographic data

Characteristics	Group A^**†**^ (***N*** = 10)	Group B^**‡**^ (***N*** = 21)	p value*
**Mean age (years)**	32.6 ± 11.1	32.4 ± 13.9	0.403
**Sex**			0.075
Women	2 (20%)	9 (43%)	
Men	8 (80%)	12 (57%)	
**Injured wrist**			0.085
Right	5 (50%)	14 (67%)	
Left	5 (50%)	7 (33%)	
**Dominant side injury**	6 (60%)	13 (62%)	0.245
**Concomitant DRF**^₤^	1 (10%)	8 (38%)	0.034*
**Time to surgery (months)**	6.6	7.4	0.445

†Patients who underwent anchor suture fixation

‡Patients who underwent tension band wiring fixation

₤DRF = distal radial fracture

*p values in bold represent statistical significance (*p* < 0.05)

**Table 2:** Case number in each radiographic fracture pattern

Gaulke†	1A	1B	1C	2A	2B	2C
**Group A (*N*= 10)**	1	2	3	2	2	
**Group B (*N*= 21)**				13	1	7

†Gaulke classification

### Surgical procedure

**Fig. 1:**
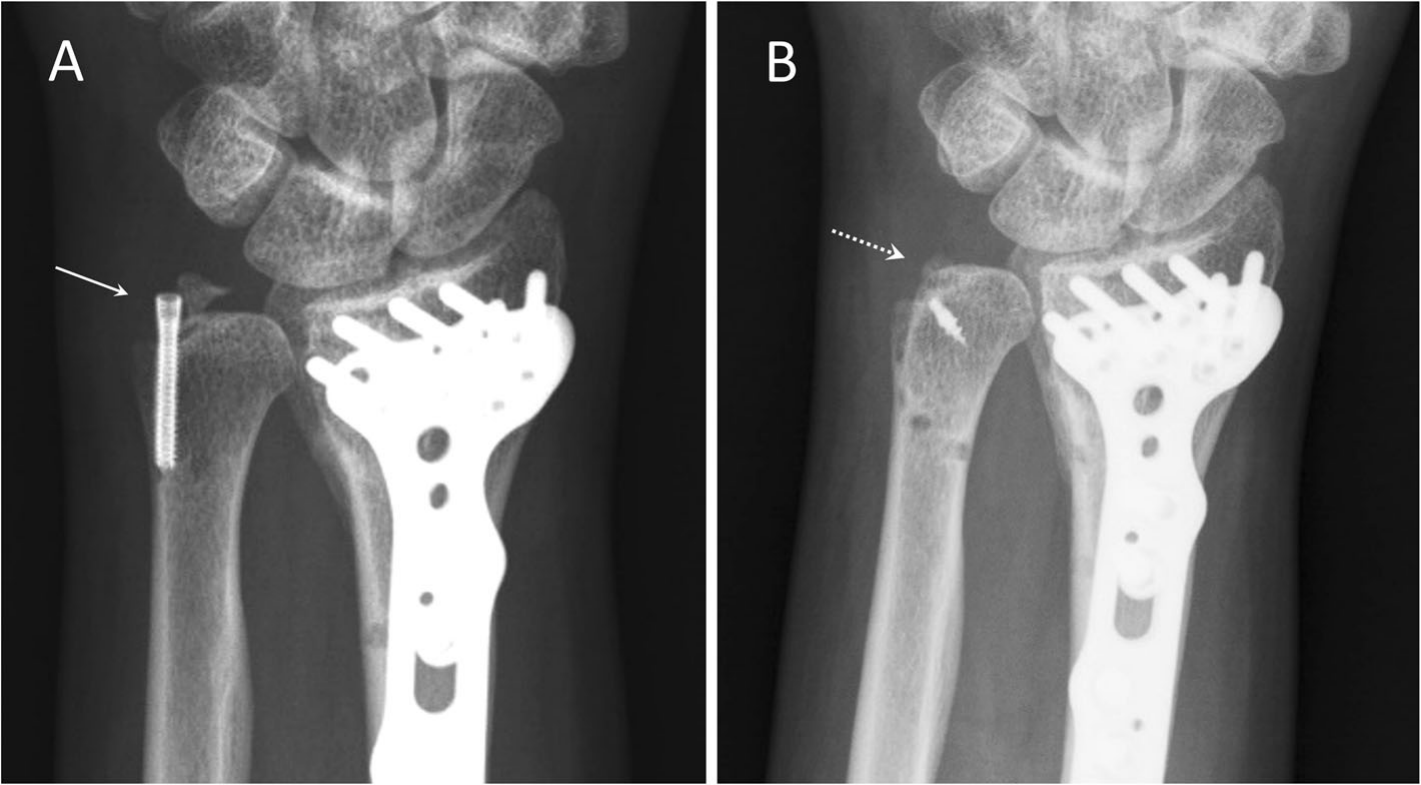
Posteroanterior radiographs of a 35-year-old male patient receiving open reduction and internal fixation for distal radioulnar fracture. **a** Ulnar styloid displacement with nonunion (arrow) at 4 months after screw fixation. **b** Osseous union (dotted arrow) at 3 months after revision fixation with anchor suture fixation

**Fig. 2:**
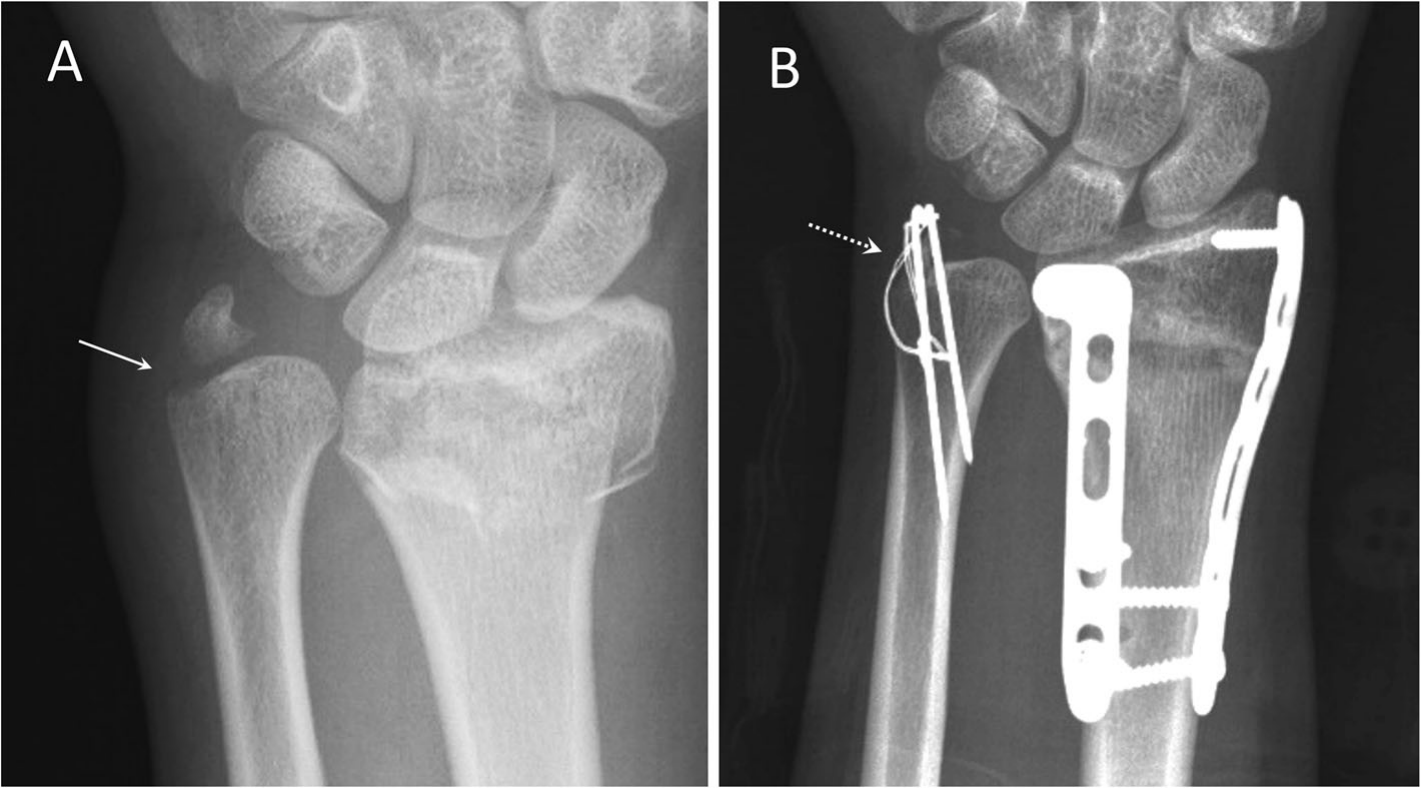
Posteroanterior radiographs of a 20-year-old female patient with distal radioulnar fractures. **a** Displaced ulnar styloid base fracture (arrow). **b** Osseous union (dotted arrow) at 3 months after surgical fixation with tension band wire

All the surgery was conducted with patients in general anesthesia and by a single surgeon. The patient was in supine position with the operated arm prepared on a hand table. A 4- to 5-cm curvilinear incision was made over the dorso-ulnar side of wrist. Gentle dissection with meticulous protection of the dorsal sensory branch of ulnar nerve (DSBUN) was performed and deepened into extensor retinaculum and joint capsule that were divided along the ulnar side of extensor carpi ulnaris tendon compartment. Once the fracture was identified, double-loaded retention suture using 2-O ethibond was passed through the soft tissue attachment on the styloid tip to facilitate subsequent maneuver traction and reduction. Additional debridement and decortication of the fracture ends were undertaken for chronic cases. For group A, a 2.0 mm MINITAC™ Ti Suture Anchor (Smith & Nephew; MA, USA) was inserted into the ulnar styloid base after predrilling (Fig. 1). The double-loaded ULTRABRAID™ Sutures were used to pass the avulsion bony fragments for fixation. The retention ethibond sutures were passing the drilling holes over the distal ulnar cortex to serve as a tension band for fixation augmentation. For group B, the styloid fracture was reduced first and then transfixed with tension band wiring using two 0.045″ Kirschner wires and cerclage wire (Fig. 2). DRUJ stability was rechecked after styloid fixation. The retention ethibond suture was used for augmentation as in group A. Fracture reduction and DRUJ congruity was confirmed by mini C arm image intensifier followed by percutaneous pinning of distal radius and ulna in neutral rotation position for temporary immobilization. With completion of styloid fixation, the retinaculum and skin were approximated with subcuticular sutures. After surgery, a short arm splint was applied for 4 weeks. Percutaneous Kirschner wire in group A was removed at 4 weeks, and rehabilitation with gentle wrist motion was started. Return to work was allowed after 3 months postoperatively. Fixation implant in group B was not removed routinely unless there was local attrition owing to pin and wire.

### Functional and radiographic assessment

Radiographic follow-up was taken the next day, at 3 months, and 2 years after surgery. Fracture union was defined according to both clinical and radiographic assessment. If the fractures were not healed at 3 months, additional radiographs were taken every 3 months thereafter until 1 year after surgery. Functional outcome measured at 2 years after surgery was collected including grip strength; range of motion of the wrist and forearm; Mayo Modified Wrist Score (MMWS); 11 items in the Quick Disabilities of the Arm, Shoulder, and Hand (QuickDASH) score; and residual wrist pain. Functional survey was calculated in. The QuickDASH with scoring from one to five was used to evaluate perceived physical function and symptoms in individuals with upper limb musculoskeletal disorders [13]. Residual pain was rated from zero to ten using visual analog scale (VAS). Complications including nonunion, infection, and impact related sequelae were reported according the review of medical records.

### Statistical analysis

Descriptive statistics were used to analyze each key variable. An independent *t* test was used in normally distributed data including patient age and time interval between trauma and fixation, the Mann-Whitney rank sum test, in numerical data that was not normally distributed (QuickDASH score). A chi-square test was used to compare categorical data including sex, injured hand and dominance, trauma chronicity, associated DRF, and complications. Statistical significance was defined as a *p* value < 0.05.

## Results

### Surgical data

A total of 31 patients of ulnar styloid fracture with DRUJ instability were reported with a mean follow-up of 2.2 years (range, 2 to 4). Radiographic classification was presented in Table 2. Two patients in group A and one patient in group B were revision cases that have failed previous surgery. In group A, all 10 patients underwent anchor suture fixation of ulnar styloid plus DRUJ pinning. In group B, all 21 patients underwent tension band wire fixation of ulnar styloid fracture; 6 of the 21 patients received simultaneous TFCC repair.

### Functional outcome

Osseous union was documented both clinically and radiographically in 29 patients (94%) including 10 in group A and 19 in group B. Functional measurement at postoperative 2 years was compared and analyzed between the two groups (Table 3). Grip strength averaged 33.0 (range, 21 to 41) kg in group A and 33.0 (range, 22 to 45) kg in group B; there was no significant difference. Evaluation of wrist motion range consisting of 4 items was compared between two groups. Supination averaged 79.0° (range, 60 to 85°) and 78.3° (range, 55 to 85°) in groups A and B respectively; pronation, 76.0° (range, 60 to 80°) and 77.1° (range, 65 to 85°); extension, 81.5° (range, 75 to 85°) and 80.2° (range, 65 to 90°); and flexion, 75.5° (range, 70 to 80°) and 76.9° (range, 70 to 85°). No significant difference was found in all 4 items of motion evaluation. Residual pain was measured with a mean VAS of 1.1 (range, 0 to 3) in group A and 0.8 (range, 0 to 2) in group B without significant difference. Functional survey in MMWS averaged 82.5 (range, 65 to 100) in group A and 84.0 (range, 75 to 95) in group; the difference was not significant. QuickDASH averaged 11.1 (range 0 to 31.8) in group A and 7.5 (range, 0 to 34.1); no significant difference was found.

**Table 3:** Functional outcome

Items	Group A (***N*** = 10)	Group B (***N*** = 21)	p value*
**Grip strength (kg)**	33.0 ± 6.7	33.0 ± 7.5	0.500
**Range of motion (degrees)**			
**Supination**	79.0 ± 7.7	78.3 ± 7.3	0.409
**Pronation**	76.0 ± 6.1	77.1 ± 5.9	0.284
**Extension**	81.5 ± 4.1	80.2 ± 7.5	0.312
**Flexion**	75.5 ± 3.1	76.9 ± 6.6	0.269
**Pain score**	1.1 ± 1.0	0.8 ± 0.8	0.197
**MMWS**	82.5 ± 10.6	84.0 ± 6.2	0.306
**QuickDASH**	11.1 ± 9.9	7.5 ± 8.2	0.143

*MMWS* Mayo Modified Wrist Score, *QuickDASH* Quick Disabilities of the Arm, Shoulder, and Hand

*p values in bold represent statistical significance (*p* < 0.01)

### Surgical complications

There was no neurovascular damage, surgical site infection, impaired wound healing, or other major surgical complications. Bone and implant-related complications were summarized in Table 4. Two patients in group B presented radiographic nonunion at latest follow-up and did not receive subsequent surgical management. Both were revision cases that had failed to previous screw fixation surgery. Residual DRUJ subluxation that was defined both clinically by positive piano key sign and radiographically by on the lateral view of latest follow-up was noted in one patient of group A and two patients of group B. Partial bony resorption of ulnar styloid process was noted on radiographs of two patients in group A and two patients in group B. Totally, there were three (30%) and six (29%) bone-related complications in groups A and B respectively; no significant difference was noted. Implant migration was noted in three patients of group B. Ten patients in group B returned to operation room for implant removal owing to local attrition. One patient in group A exhibited pin tract discharge and subsided after pin removal. Totally, there were 1 (10%) and 10 (48%) implant-related complications in groups A and B respectively; the difference was significant with a *p* value of 0.021.

**Table 4:** Surgical complications

Items	Group A (***N*** = 10)	Group B (***N*** = 21)	p value*
**Patients with complications**	4 (40%)	10 (48%)	0.194
**Bone related**	3 (30%)	6 (29%)	0.469
Nonunion	0	2	
DRUJ subluxation	1	2	
Bone resorption	2	2	
**Implant related**	1 (10%)	10 (48%)	0.021*
Migration	0	3	
Removal	0	10	
Infection	1	0	

*p values in bold represent statistical significance (*p* < 0.01)

## Discussion

There has long been conflict regarding clinical relevance in fixation of ulnar styloid fractures. A recent meta-analysis revealed functional scores favoring distal radius fractures without concomitant ulnar styloid fractures while the outcome difference was not significant in patients with and without ulnar styloid fractures [14]. In literature review, most of those studies anecdotally evaluated ulnar styloid fractures based on the outcomes of concomitant distal radius fractures and commonly introduced bias assuming more complicated presentation in surgical treated ulnar styloid fractures than non-surgical cases to reach a conclusion of no significant difference [15]. Besides, surgical concerns existed almost uniquely in styloid base fractures owing to both fixation feasibility [7] and cadaveric demonstration [16]. However, wrist function could be adversely affected in several additional ways such as fracture line extension, disruption of secondary stabilizers, and associated TFCC injury [17]. Principles of management may include anatomical restoration, fracture fixation, and TFCC repair. Our previous study suggested early fixation of ulnar styloid fractures to yield better outcome than late surgery [18].

In comparing patient characteristics between two groups, significant difference was only noted in the item of concomitant DRF; significantly higher incidence was noted in group B than group A (*p* = 0.034). It may reasonably correspond to the common concerns of ulnar styloid base fracture in surgically treated cases as well as cadaveric research. In contrary, presentation of styloid tip avulsion in fracture orientation and fragmentation was insufficiently defined and generally overlooked even if there was concurrent DRF and DRUJ instability. There were nine cases of concomitant DRFs in our study. All except one were chronic ulnar styloid fractures, which underwent subsequent surgical fixation owing to persistent ulnar wrist pain and decreased range of motion while the concomitant DRFs had been well reduced and fixed with locking plates. The case with acute injury was presented in Fig. 2; surgical fixation of ulnar styloid base fracture was performed simultaneously since remarkable DRUJ laxity was noted following double plating of the DRF. In addition to the commonly mentioned styloid base fracture, we presented difference fracture patterns and proposed an innovated fixation option with favorable treatment outcomes.

Being a morphological categorization, Gaulke classification was proposed to further divide the ulnar styloid fractures according to fracture location and orientation [12].

**Fig. 3:**
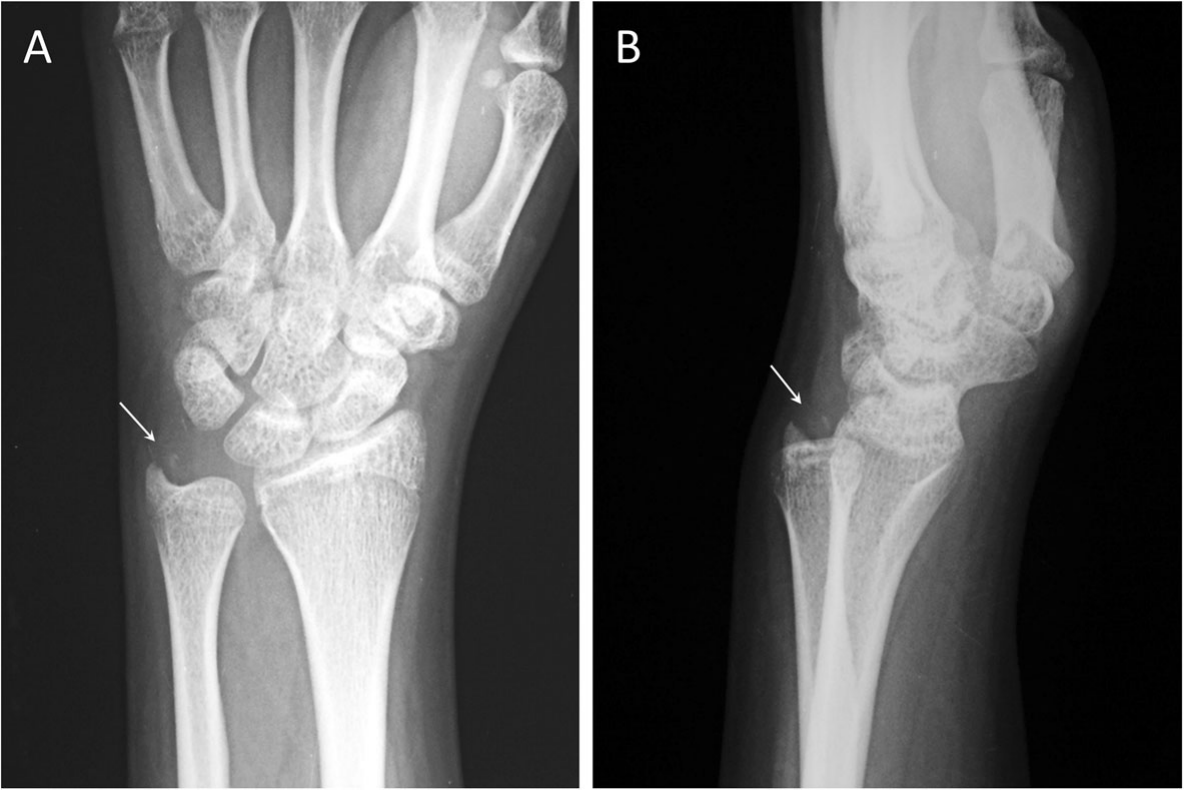
Radiographs of a 47-year-old female patients. **a** Posteroanterior projection showed small ulnar styloid tip fracture (arrow). **b** Lateral projection demonstrated diagonal ulnar side ascending course of the fracture (arrow) with subluxed distal radioulnar joint

Fracture displacement was found to independently affect healing of ulnar styloid in different fracture patterns. Given a wide range of nonunion rate in ulnar styloid fractures [19], Gaulke classification established a morphological categorization with predictive value to serve guidance for treatment. In our series, four cases in group A were classified as B Gaulke B types; two were IB and two, IIB. The remaining six cases included one IA, three IC, and two 2A. However, the fracture pattern in the two cases classified as IIB was identified from lateral view (Fig. 3) instead of anteroposterior view that was originally defied in Gaulke classification. Owing to the diversity of fracture patterns, we emphasize that meticulous radiographic survey in different projections is crucial to facilitate correct diagnosis and proper management. On the other hand, the majority of group B were categorized as 2A (13 cases) and 2C (seven cases); only one case was 2B. In the index surgery for group A, fixation using small anchors allowed those tiny fracture fragments in group A to be sutured and fixed back to distal ulna and yielded comparable outcome to tension band wiring of styloid base fractures in group B. In spite of slightly better functional results based on MMWS and QuickDASH in groups B than A, the difference was insignificant.

Excision of bony fragment plus transosseous tissue repair has been recommended in two studies [6, 8] in symptomatic ulnar styloid avulsion fractures with small bony fragments. A recently published cadaveric study further documented the location and importance of ulnar collateral ligament around the ulnar styloid [20] in addition to TFCC and capsular insertion traditionally mentioned in the literature. Refined small anchors with double-loaded ultra-braid suture allowed avulsion bony fragments to be sutured back feasibly and efficiently while traditional transosseous suture techniques still could serve as an augmentation by securing regional tissue and capsule to the distal ulna.

The incidence of surgical complication was comparable for both groups. Nonunion and residual DRUJ instability was slightly more common in group B. Radiographic resorption of ulnar styloid fragments was observed in both groups. None of those patients presented symptomatic instability and received revision surgery. Implant-related complication rate was significantly higher in group B (*p* = 0.021). Symptoms of implant irritation or migration with subsequent removal surgery were commonly seen in group B, but none was in group A. Local discharge around the percutaneous Kirschner wire for DURJ fixation was noted only in one patient of group A and soon subsided with wire removal. None in group A exhibited migration or loosening of suture anchor and underwent secondary surgery.

The main limitations of this study include small cohort number and heterogeneity of fracture characteristics. Analysis of trauma mechanism with correlation to functional outcome is not allowed based on retrospective reviews of medical records. In addition, a follow study is necessary to elucidate the long-term effect of the index surgery on wrist function and DRUJ sequelae.

## Conclusions

Surgical fixation in symptomatic ulnar styloid fractures with DRUJ instability is a feasible option with encouraging outcomes. Functional results in anchor-suture group and tension-band-wire group are comparable while the implant-related complication rate is higher in the latter. Small-sized suture anchors with double-loaded ultra-braid suture may allow fixation of avulsion bony fragments with limited surgical complication.

## Data Availability

The datasets generated during the current study are available from the corresponding author on reasonable request.

## Abbreviations

DRUJ: Distal radioulnar joint
TFCC: Triangular fibrocartilage complex
DRF: Distal radial fracture
DSBUN: Dorsal sensory branch of ulnar nerve
MMWS: Mayo Modified Wrist Score
QuickDASH: Quick Disabilities of the Arm, Shoulder, and Hand
VAS: Visual analog scale
